# Anti-Fibrosis Effect of Scutellarin via Inhibition of Endothelial–Mesenchymal Transition on Isoprenaline-Induced Myocardial Fibrosis in Rats

**DOI:** 10.3390/molecules191015611

**Published:** 2014-09-29

**Authors:** Hao Zhou, Xiao Chen, Lingzhi Chen, Xi Zhou, Gaoshu Zheng, Huaiqin Zhang, Weijian Huang, Jiejie Cai

**Affiliations:** 1 Department of Cardiology, The First Affiliated Hospital of Wenzhou Medical University, Wenzhou 325000, China; E-Mails: wyzh66@126.com (H.Z.); zhenggaoshu@163.com (G.Z.); zhanghuaiqin@126.com (H.Z.); huangweijian@hosp1.ac.cn (W.H.); 2 Department of Cardiology, Ningbo First Hospital, Ningbo 315000, China; E-Mail: cx6176@gmail.com; 3 Clinical LaboratoryDepartment, Wenzhou Central Hospital, Wenzhou 325000, China; E-Mail: clzwz@sina.com; 4 Coronary Care Unit, The First Affiliated Hospital of Wenzhou Medical University, Wenzhou 325000, China; E-Mail: zhouxihw@gmail.com; 5 Intensive Care Unit, The First Affiliated Hospital of Wenzhou Medical University, Wenzhou 325000, China

**Keywords:** scutellarin, cardiac fibrosis, EndMT, angiogenesis, notch pathway

## Abstract

Scutellarin (SCU) is the major active component of breviscapine and has been reported to be capable of decreasing myocardial fibrosis. The aim of the present study is to investigate whether SCU treatment attenuates isoprenaline-induced myocardial fibrosis and the mechanisms of its action. Rats were injected subcutaneously with isoprenaline (Iso) to induce myocardial fibrosis and rats in the SCU treatment groups were intraperitoneally infused with SCU (10 mg·kg^−1^·d^−1^ or 20 mg·kg^−1^·d^−1^, for 14 days). Post-treatment, cardiac functional measurements and the left and right ventricular weight indices (LVWI and RVWI, respectively) were analysed. Pathological alteration, expression of type I and III collagen, Von Willebrand factor, α-smooth muscle actin, cluster of differentiation-31 (CD31), and the Notch signalling proteins (Notch1, Jagged1 and Hes1) were examined. The administration of SCU resulted in a significant improvement in cardiac function and decrease in the cardiac weight indices; reduced fibrous tissue proliferation; reduced levels of type I and III collagen; increased microvascular density; and decreased expression of α-smooth muscle actin and increased expression of CD31, Notch1, Jagged1 and Hes1 in isoprenaline-induced myocardial fibrosis in rats. Our results suggest that SCU prevents isoprenaline-induced myocardial fibrosis via inhibition of cardiac endothelial-mesenchymal transition potentially, which may be associated with the Notch pathway.

## 1. Introduction

Myocardial fibrosis plays a major role in the occurrence and development of a number of different heart diseases. Studies have shown that the occurrence of chronic heart failure is closely related with myocardial fibrosis, with this fibrosis leading to irreversible heart damage and thereby altering heart function from compensated to decompensated. Thus, prevention of myocardial fibrosis could theoretically improve cardiac function and even reverse ventricular remodelling. 

EndoMT is a complex biological processin by which endothelial cells lose their specific markers and acquire a mesenchymal or myofibroblastic phenotype and express mesenchymal cell products such as α smooth muscle actin (α-SMA) and type I collagen [1]. The effects of EndMT in embryonic development, tissue regeneration, wound healing, and neoplasia have been thoroughly investigated, and, recently, EndMT has also been reported to play a role in fibrosis in various tissues, including the lungs, kidneys and the mesenteric and coronary arteries. Zeisberg *et al.* recently reported on EndMT in heart fibrosis [2], which has opened a new chapter of myocardial fibrosis mechanism research. 

Notch is a highly conserved signalling pathway between adjacent cells that mediates key cell fate decisions during proliferation, differentiation, and apoptosis. Aberrant Notch signalling can cause abnormal heart development, and recent studies have shown that Notch signalling plays an important role in anti-fibrosis treatment. In transgenic mice overexpressing the Notch ligand Jagged1, Notch was found to inhibit the development of cardiomyocyte hypertrophy and transforming growth factor-β/connective tissue growth factor-mediated cardiac fibrosis [3].

**Figure 1:**
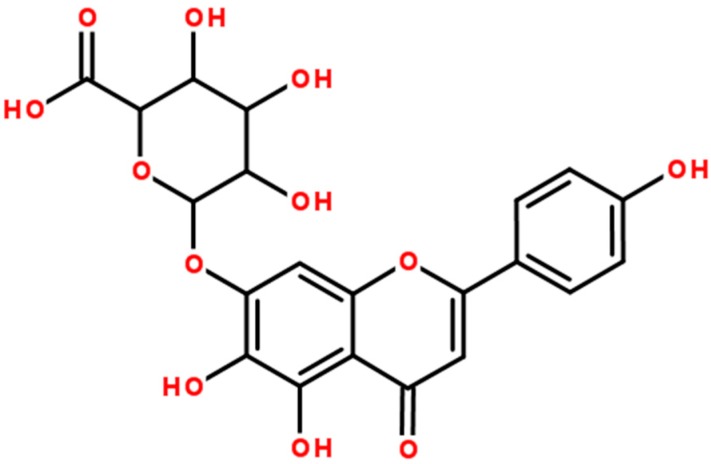
Scutellarin (SCU, 5,6,4-trihydroxyflavone-7-O-glucoronide).

Scutellarin (SCU, 5,6,4-trihydroxyflavone-7-O-glucoronide, Figure 1) is a flavone and the major active component of breviscapine. SCU is a herbal medicine widely used for the treatment of cerebrovascular diseases and has been demonstrated to have anti-apoptotic, antioxidant, anti-inflammatory, and calcium channel antagonist properties in the researches of nervous system disease. The studies related to the physicochemical property and bioavailability of SCU also have been done to ensure the optimal utilization of SCU [4,5]. Recently, SCU is found to display beneficial cardiovascular effects, including vasorelaxant effects [6], inhibition of high glucose-mediated vascular inflammation [7], angiogenesis-promoting effects [8], and alleviation of cardiac dysfunction and interstitial fibrosis [9]. However, the specific mechanism behind anti-fibrosis effect is still unclear.

On the basis of these observations, the aims of the present study were to investigate whether SCU treatment reduces isoprenaline (Iso)-induced myocardial fibrosis in a rat model, to examine the effects of SCU on EndMT and the expression of Notch signalling proteins, and to investigate the relationship between these changes and myocardial fibrosis.

## 2. Results and Discussion

### 2.1. SCU Improves Cardiac Function

Iso was found to induce significant decreases in LVSP, +dp/dtmax and −dp/dtmax, and an increase in LVEDP (*p* < 0.01), whereas treatment with SCU attenuated these Iso-induced changes in ventricular function (*p* < 0.05; Table 1).

**Table 1:** Effect of scutellarin (SCU) on the cardiac functional index in an isoprenaline (Iso)-induced myocardial fibrosis rat model ($\bar{x}$ ± s; n = 8).

Group	LVSP/mmHg	LVEDP/mmHg	+dp/dtmax/mmHg/s	−dp/dtmax/mmHg/s
NS control	139.4 ± 12.6	−7.70 ± 8.35	10257.0 ± 2500.7	−9082.2 ± 2239.7
Iso	108.8 ± 11.6 **	20.34 ± 8.21 **	3547.3 ± 1651.0 **	−3095.3 ± 1249.2 **
Iso + low dose SCU	124.5 ± 14.7 ^#^	1.55 ± 3.07 ^##^	6015.5 ± 1995.7 ^#^	−5241.2 ± 1492.9 ^#^
Iso + high dose SCU	136.9 ± 11.7 ^##^	−3.67 ± 4.09 ^##^	9323.6 ± 2409.7 ^#^	−7974.8 ± 2683.7 ^##^

** *p* < 0.01 *vs*. control group; ^#^ *p* < 0.05, ^##^ *p* < 0.01 *vs*. Iso group.

### 2.2. SCU Treatment Results in Decreased LVWI and RVWI

LVWI and RVWI were significantly higher in the Iso-treated group than in the control group (*p* < 0.01). Treatment with both high-dose and low-dose SCU resulted in significant decreases in LVWI and RVWI compared to the Iso-treated group (*p* < 0.01). However, no significant differences were observed between the two SCU-treated groups (Table 2).

**Table 2:** Effects of scutellarin (SCU) on the weight index in an isoprenaline (Iso)-induced myocardial fibrosis rat model ($\bar{x}$ ± s; n = 10).

Group	LVWI/mg·g^−1^	RVWI/mg·g^−1^
NS control	2.46 ± 0.20	0.63 ± 0.08
Iso	3.10 ± 0.30 **	0.93 ± 0.15 **
Iso + low dose SCU	2.65 ± 0.34 ^##^	0.74 ± 0.13 ^##^
Iso + high dose SCU	2.51 ± 0.28 ^##^	0.64 ± 0.08 ^##^

** *p* < 0.01 *vs.* control group; ^##^ *p* < 0.01 *vs.* Iso group.

### 2.3. Histopathological Observations of the Myocardium

**Figure 2:**
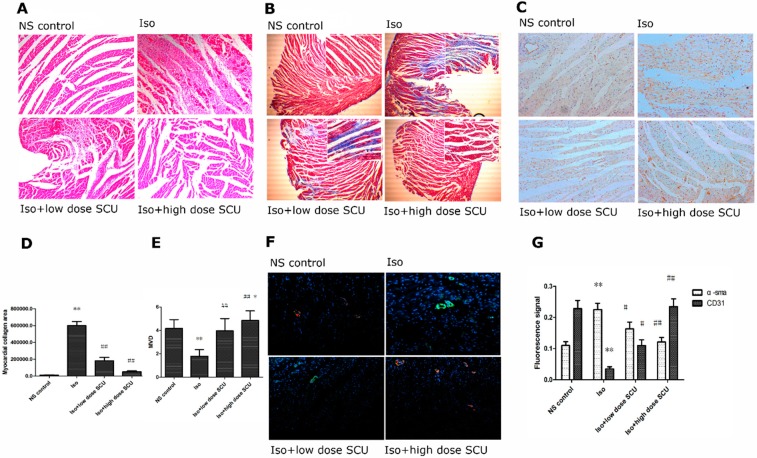
(**A**) Haematoxylin and eosin staining of the left ventricular myocardium. Magnification ×200; (**B**,**D**) Myocardial collagen areas in the four groups. The cardiomyocytes were stained in red, fibrous tissues were stained in blue. Magnification ×40. Photomicrographs in the top right corner were taken at the magnification of ×200; (**C**,**E**) Effects of SCU on microvascular density (MVD) in Iso-induced myocardial fibrosis rat models. Magnification ×200; (**F**,**G**) Fluorescence signals of α-sma and CD31 in the four groups. Cells were immunostained with antibodies against α-sma (myofibroblastic phenotype; green) and CD31 (endothelial phenotype; red), and the nuclei were labelled using DAPI dihydrochloride (blue). Magnification ×200. * *p* < 0.05, ** *p* < 0.01 *vs.* control group; ^#^ *p* < 0.05, ^##^ *p* < 0.01 *vs.* Iso group.

Heart tissues from Iso-treated rats showed widespread fibrous tissue proliferation, myocardial structure disorder, myocardial hypertrophy, vacuolization and leukocyte infiltration compared with the control group. Treatment with SCU produced a marked improvement in Iso-induced fibrous tissue proliferation, subendocardial necrosis, and leukocyte infiltration (Figure 2A).

### 2.4. SCU Treatment Results in Decreased Myocardial Fibrosis

Stained myocardium from Iso-treated rats showed widespread blue fibrous tissue (*p* < 0.01, Figure 2B,D) and significantly increased concentrations of type I and type III collagen in the tissue homogenate compared with the control group (*p* < 0.01, Table 3). Treatment with both high-dose and low-dose SCU significantly decreased Iso-induced fibrous tissue proliferation and concentrations of type I and type III collagen (*p* < 0.01 for all) (Figure 2B,D, Table 3).

**Table 3:** Expression of type I collagen and type III collagen in the four groups ($\bar{x}$ ± s).

Group	n	Type I collagen/ng·mL^−1^	Type III collagen/ng·mL^−1^
NS control	10	2.37 ± 0.86	2.36 ± 1.04
Iso	9	7.54 ± 1.67 **	5.68 ± 1.31 **
Iso + low dose SCU	10	5.73 ± 1.86 ^##^	4.12 ± 0.94 ^##^
Iso + high dose SCU	10	2.79 ± 0.76 ^##^	3.00 ± 0.86 ^##^

** *p* < 0.01 *vs.* control group; ^##^ *p* < 0.01 *vs.* Iso group.

### 2.5. SCU Treatment Increases the Microvascular Density (MVD)

The MVD in the Iso-treated group was significantly lower than in the control group (*p* < 0.01). Treatment with high-dose SCU resulted in a significant increase of MVD compared with the Iso-treated group (*p* < 0.01). Interestingly, the MVD in the high-dose SCU group was even higher than in the control group (*p* < 0.05). Low-dose SCU treatment also resulted in an obvious increase in MVD compared with the Iso-treated group (*p* < 0.01), but this was not as significant as for the high-dose group (Figure 2C,E).

### 2.6. Immunofluorescence Observations of the Myocardium

Vascular endothelial cells from Iso-treated rats showed an increase in the intensity of α-sma fluorescence and a decrease in the intensity of CD31 fluorescence compared with the control group (*p* < 0.01). Treatment with SCU significantly attenuated the Iso-induced changes in intensity of α-sma and CD31 fluorescence (*p* < 0.05; Figure 2F,G).

### 2.7. SCU Treatment Leads to an Increase in the Expression of CD31 Protein and a Decrease in the Expression of α-sma Protein in Isoprenaline-Induced Myocardial Fibrosis in Rats

**Figure 3:**
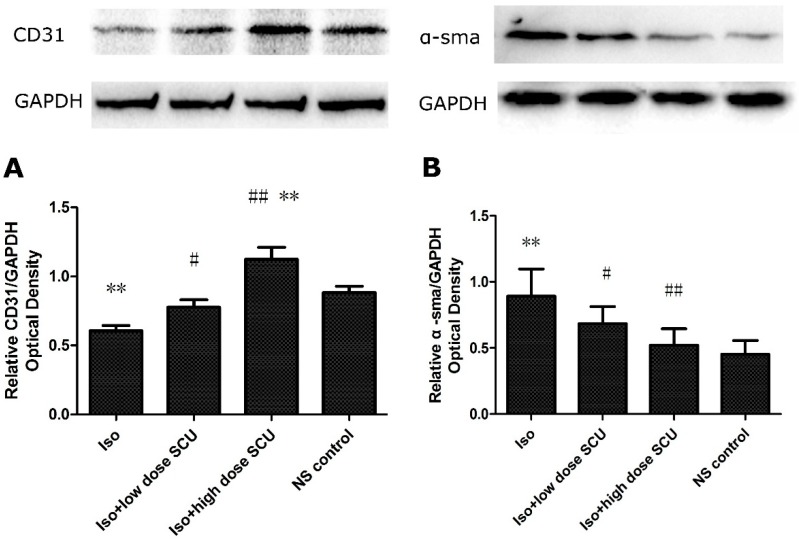
Western blot analyses for CD31 and α-sma in the four groups. (**A**) The expression of CD31 protein ($\bar{x}$ ± s; n = 12); (**B**) The expression of α-sma protein ($\bar{x}$ ± s; n = 8).

The expression of CD31 protein and α-sma protein, as determined by western blotting was significantly lower and higher in the Iso-treated group than in the control group respectively (*p* < 0.01; Figure 3). Treatment with SCU resulted in an increase in the expression of CD31 protein and a decrease in the expression of α-sma protein compared with the Iso-treated group (*p* < 0.05; Figure 3). Interestingly, the expression of CD31 protein in the high-dose SCU group was even higher than in the control group (*p* < 0.01; Figure 3A).

** *p* < 0.01 *vs.* control group; ^#^ *p* < 0.05, ^##^ *p* < 0.01 *vs.* Iso group. GAPDH, glyceraldehyde 3-phosphate dehydrogenase. Iso, isoprenaline; SCU, scutellarin.

**Figure 4:**
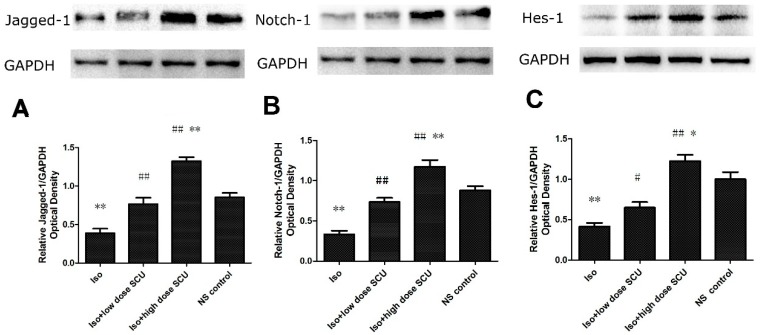
Western blot analyses for Notch1, Jagged1, and Hes1 in the four groups ($\bar{x}$ ± s; n = 12). (**A**) The expression of Jagged1 protein; (**B**) The expression of Notch1 protein; (**C**) The expression of Hes1 protein. Notch 1, Jagged1 and Hes1 protein expression levels were significantly lower in the Iso-treated group than in the control group. Treatment with high-dose SCU resulted in significant increases of these proteins compared with both the Iso-treated and control groups. Low-dose SCU treatment also resulted in an obvious increase in these proteins compared with the Iso-treated group, but was not as significant as for high-dose SCU treatment.

* *p* < 0.05, ** *p* < 0.01 *vs.* control group; ^#^ *p* < 0.05, ^##^ *p* < 0.01 *vs.* Iso group. GAPDH, glyceraldehyde 3-phosphate dehydrogenase. Iso, isoprenaline; SCU, scutellarin.

### 2.8. SCU Increases the Expression of Jagged1, Notch 1, and Hes1 Proteins

Jagged1, Notch 1 and Hes1 protein expression levels, as determined by western blot analyses, were significantly lower in the Iso-treated group than in the control group (*p* < 0.01). Treatment with high-dose SCU resulted in significant increases of these proteins compared with both the Iso-treated and control groups (*p* < 0.01 for both). Moreover, a significant increase in these proteins was observed after low-dose SCU treatment compared with the Iso-treated group (*p* < 0.05), but this was not as significant as for the high-dose group (Figure 4).

### 2.9. Discussion

In our study, we found the Iso-treated rats showed lower cardiac function, higher LVWI and RVWI, impaired cardiac structure, increased fibrous tissue proliferation and levels of type I and III collagen compared with the control group, and the administration of SCU alleviated these changes. Our results demonstrate the anti-fibrosis effect of SCU in isoprenaline-induced myocardial fibrosis in rats. The improvement of myocardial fibrosis was more obvious in rats administrated with high-dose SCU (20 mg/kg/d) than those received 10 mg/kg/d, suggesting the anti-fibrosis effect of SCU may be dose related.

In recent years, numerous studies have shown that EndMT plays an important role in fibrosis. Zeisberg *et al.* recently reported on EndMT in heart fibrosis [1]. After mice underwent aortic banding to induce myocardial fibrosis, they found specific labeled endothelial cells scattered throughout the fibrotic area expressed multiple myofibroblastic phenotypes. In vitro, acquisition of a spindle-shaped morphology upon transforming growth factor-β1 (TGF-β1), which is known to promote and induce EndMT [10,11], endothelial cells also gained a fibroblast-like phenotype, whereas untreated control cells preserved their endothelial cell shape and phenotype. During EndMT, the biological function of edothelial cells also get close to fibroblasts.

EndMT is a complex biological process that endothelial cells lose their endothelial features and gain mesenchymal or myofibro-blastic properties. Goumans *et al.* recently pointed out that in cardiac fibrosis, EndoMT represents the most important contributor to the generation of fibrotic tissue [12].

On the one hand, when endothelial cells are damaged, they secrete increased amounts of TGF-β, angiotensin-II, and endothelin-1, which are known to induce myocardial fibrosis, whereas the secretion of nitric oxide and bradykinin, which are known to suppress fibrosis, is decreased. Meanwhile, loss of microvascularity is also known to be involved in the process of tissue fibrosis [13]. However, EndMT is associated with endothelial cell damage and decreases in microvessel density.

On the other hand, in the heart, as well as in these other organs, the predominant cellular mediators of fibrosis are thought to be (myo)fibroblasts [12]. Myofibroblasts, the activated form of cardiac fibroblasts, are capable of secreting extracellular matrix components such as collagen, fibronectin, and laminin to promote the development of fibrosis and about 27%~35% of myofibroblasts were of endothelial origin through EndMT [2]. 

In the present study, we found that vascular endothelial cells from Iso-treated rats showed decreased CD31 protein and MVD, and increased expression of type I/III collagen and α-sma compared with control rats, which indicates attenuated endothelial characteristic and enhanced fibroblast property, suggesting the EndMT in Iso-induced myocardial fibrosis. While treatment with SCU attenuated these effects, suggesting that SCU treatment improves myocardial fibrosis via inhibition of EndMT potentially. Interestingly, the expression of CD31 protein and MVD in SCU groups was increased, and in the high-dose SCU group the expression of CD31 protein and MVD was even higher than in the control group. Thus, we speculate that SCU might have angiogenesis-promoting effect in intervening in myocardial fibrosis. And, the angiogenesis-promoting effect of SCU has been demonstrated recently by Ma *et al.*, and this effect may be dose related [8]. Indeed, further studies should be done in the future to confirm the angiogenesis-promoting effect of SCU in intervening in myocardial fibrosis and investigate the mechanisms of its action, which may provide a new scope for the treatment of cardiac fibrosis.

The Notch signalling pathway involves the Notch-1/2/3/4 receptors and their transmembrane ligands (Jagged-1/2 and agged-1/3/4). Upon ligand binding, the Notch intracellular domain is released by the γ-secretase complex, migrates into the nucleus, and interacts with transcriptional repressors, thereby regulating target genes such as the basic helix-loop-helix proteins Hes and Hey.

A number of previous studies have shown that the Notch signalling pathway regulates myocardial fibrosis. In transgenic mice overexpressing the Notch ligand Jagged1, Notch was found to inhibit the development of cardiomyocyte hypertrophy and TGF-β/connective tissue growth factor-mediated cardiac fibrosis [3]. Moreover, *in vitro* studies have demonstrated that Notch signalling influences the differentiation of fibroblasts into myofibroblasts [14,15]. 

In this study, the protein expression of Notch 1, Jagged1, and Hes1 proteins in the Iso-treated group were significantly lower than in the control group, and treatment with SCU was found to increase the expression of these proteins to the point when, in the high-dose SCU group, it was even higher than in the control group. These results suggest that suppression of myocardial fibrosis by SCU may be associated with the Notch pathway. However, the evidence is limited and further study should be done. 

In myocardial fibrosis, endothelial cells provide sources for fibroblasts via EndMT. TGF-β is often used to to promote and induce EndMT, and Notch signalling has been previously demonstrated to inhibit TGF-β-induced EndMT and myocardial fibrosis [3]. Furthermore, Previous research found that TGF-β1 could increase the expression of α-sma and down-regulate Notch receptors 1, 3, and 4 in cardiac fibroblasts, and that Notch signalling inhibitors increased the expression of α-sma [15]. In this study, the Iso-treated rats showed increased expression of α-sma and decreased expression of CD31, Notch1, Jagged1 and Hes1 compared with control rats, while the administration of SCU resulted in decreased expression of α-sma and increased expression of CD31, Notch1, Jagged1 and Hes1, that was, SCU restrained EndMT and up-regulated of Notch signalling in our rat model. According to this, we made a hypothesis that the probable molecular mechanism of SCU inhibiting the EndMT in myocardial fibrosis may be associated with the Notch pathway. And further studies should assess how Notch pathway proteins are interlinked and cooperated to mediate the effects of SCU on EndMT.

## 3. Experimental Section 

### 3.1. SCU Preparation Method 

SCU (Shanghai Boyun Biotech, Shanghai, China, 200 mg) was diluted with water for injection (100 mL) and the pH value was adjusted with weak alkaline solutions such as sodium bicarbonate and sodium hydroxide until completely dissolved. SCU solution is not very stable [4], thus, the solutions should be used immediately after preparation, or stored at −20 °C.

### 3.2. Animals and Treatments

Forty male SD rats (weight, 200–220 g; age, approximately 6 weeks) were supplied by Wenzhou Medical University Laboratory Animal Centre (Wenzhou City, Zhejiang Province, China). The animals were housed individually in cages under hygienic conditions and placed in a controlled environment with a 12 h (light)–12 h (dark) cycle at 22 ± 3 °C and 45% ± 10% humidity for 7 days before the initiation of the experiments. The animals were allowed a standard commercial diet (Laboratory Animal Centre of Zhejiang Province, Hangzhou, China) and tap water. The study was approved by the institutional research ethics committee of Wenzhou Medical University (protocol # wydw2013-0054).

At the start of the study, the rats were randomly divided into four groups (10 animals in each group): (1) myocardial fibrosis model (Iso) group; (2) low-dose SCU group (10 mg/kg/d); (3) high-dose SCU group (20 mg/kg/d); and (4) control group. On day 1–7, rats were subcutaneously injected with Iso (5 mg/kg body mass^−1^, Sigma, St. Louis, MO, USA) to induce experimental myocardial fibrosis according to the related literature [16], while rats in the control group were subcutaneously injected with normal saline. Subsequently, from day 2 to day 15, rats in the low-dose and high-dose SCU groups were intraperitoneally infused with SCU 10 mg/kg/d and 20 mg/kg/d, respectively. The method of administration and dosage were referenced to previous researches [8,17].

### 3.3. Methods

#### 3.3.1. Cardiac Functional Measurements

The rats were anaesthetised using 1% pentobarbital sodium (40 mg·kg^−1^), and lack of limbs pinch reflex indicated the surgical anaesthesia sufficient for operation. The right carotid artery was separated to allow insertion of a catheter containing 0.05% heparin saline to the left ventricle, and the other end of the catheter was connected to a PowerLab polygraph recorder (AD Instruments, Castle Hill, Australia) to record the changes of the left ventricular mean systolic pressure (LVSP), left ventricular end diastolic pressure (LVEDP), and the maximum rate of change in left ventricular pressure (+dp/dtmax, −dp/dtmax).

#### 3.3.2. Left Ventricular Weight Index (LVWI) and Right Ventricular Weight Index (RVWI)

After being humanely euthanized by bleeding, precooled saline (4 °C) was infused into the left ventricle of the rats until the heart and kidney paled. The heart was rapidly excised and rinsed in cold normal saline. Subsequently, the left and right ventricles were separated and weighed, and the left and right ventricular weight indices (LVWI and RVWI) were calculated as the left and right ventricular free wall mass (mg) divided by body mass (g), respectively.

#### 3.3.3. Haematoxylin and Eosin Staining of the Left Ventricular Myocardium

Cardiac apex samples of the left ventricle were fixed in 4% buffered paraformaldehyde solution and embedded in paraffin. Paraffin sections (4-μm thick) were stained with haematoxylin and eosin (Beyotime Institute of Biotechnology, Shanghai, China). The sections were examined by light microscopy (Nikon Corporation, Tokyo, Japan) and photomicrographs were taken at ×200 magnification.

#### 3.3.4. Masson Trichrome Staining of the Left Ventricular Myocardium

Paraffin sections were stained with Masson trichrome (GenMed Scientifics Inc., Boston, MA, USA), resulting in cardiomyocytes staining red and fibrous tissue staining blue. The sections were examined using light microscopy, photographs were taken at the magnification of ×40 and ×200. Five non-repeating visual fields, magnification ×200, were randomly selected, myocardial collagen areas were measured using Image-Pro Plus (Media Cybernetics, Inc., Bethesda, MD, USA) and the areas were averaged.

#### 3.3.5. Enzyme-Linked Immunoassay (ELISA) for Type I and III Collagen

A piece of the left ventricular myocardium (100 mg) was cut into smaller pieces, added to 1 mL phosphate buffered saline (PBS) (pH 7.4), and vortexed on ice. After centrifugation at 3000 rpm for 20 min, the supernatant was separated and the concentrations of type I and III collagen were measured using ELISA (Shanghai Boyun Biotech) according to the manufacturer’s protocol.

#### 3.3.6. Immunohistochemistry

Von Willebrand factor was used as a marker of vascular endothelial cells to indicate blood vessels. Endogenous peroxidase activity was blocked with 3% methanol-H_2_O_2_. Nonspecific sites were blocked with 5%–10% goat serum before incubation with the primary antibody (Anti-Von Willebrand factor antibody, Abcam, Cambridge, UK, 1:600) in 0.1 M PBS overnight at 4 °C. After washing, reagents 1 and 2 of the Polymer HRP Detection System for rabbit primary antibodies (Zhongshan Jinqiao, Beijing, China) were added and washed sequentially, and developed with horseradish peroxidase and diaminobenzidine chromogen (Zhongshan Jinqiao). The blood vessels were observed at ×100 and ×400 magnification before being imaged and counted at ×200 magnification. Stained single cells or cell clusters clearly separated from the surrounding vessels, cardiomyocytes, and other tissues were regarded as microvessels. The number of these microvessels was calculated as the average microvascular density (MVD) in five randomly selected non-repeating visual fields.

#### 3.3.7. Immunofluorescence Methods (IFA)

CD31 is a member of the immunoglobulin superfamily, makes up a large portion of endothelial cell intercellular junctions and is likely involved in leukocyte transmigration, angiogenesis, and integrin activation. Here, a anti-CD31 antibody was used to label endothelial cells. And, α-SMA is the major morphological characteristic of myofibroblasts. For IFA, endogenous peroxidase activity was blocked with 3% methanol-H_2_O_2_, and the nonspecific sites were treated with 10% foetal bovine serum. A mix of primary antibodies (anti-α-smooth muscle actin [sma] antibody, Wuhanboshide, Wuhan, China, 1:100; anti-cluster of differentiation [CD]31 antibody, Santa Cruz Biotechnology, Inc., SantaCruz, CA, USA, 1:30) was added to the sections and incubated overnight at 4 °C. After washing, a mix of secondary antibodies (DyLight 488 AffiniPure Goat Anti-Mouse IgG(H+L) and DyLight 594 AffiniPure Goat Anti-Rabbit IgG(H+L), EarthOX, San Francisco, CA, USA, 1:300) and DAPI staining solution (Beijing Leagene Biotechnology, Beijing, China) were added sequentially, followed by washing. Photographs were taken at ×200 magnification with a fluorescence microscope (Nikon Corporation, Tokyo, Japan) and processed with Image-Pro Plus. Cells were immunostained with antibodies against α-sma (myofibroblastic phenotype; green) and CD31 (endothelial phenotype; red), and the nuclei were labelled by DAPI dihydrochloride (blue).

#### 3.3.8. Western Blot

Equal amounts of the samples (50 µg) were loaded on gels for sodium dodecyl sulphate-polyacrylamide gel electrophoresis and transferred to polyvinylidene fluoride membranes (Beyotime Institute of Biotechnology). The membranes were blocked with 5% skim milk and incubated with the primary antibodies (anti-CD31 antibody, Santa Cruz Biotechnology, Inc., 1:1,000; anti-α-smooth muscle actin [sma] antibody, Wuhanboshide, China, 1:1000; anti-notch1 antibody, Cst, Danvers, MA, USA, 1:1000; anti-Jagged1 antibody, Cst, USA, 1:1000; anti-Hes1 antibody, Abcam, Cambridge, UK) overnight at 4 °C. Next, the membranes were incubated with the secondary antibody (HRP-conjugated goat anti-rabbit IgG(H+L), Bioworld Technology, Nanjing, China, 1:5000–1:20,000) for 2 h at room temperature. Immunoreactive bands were detected using Chemiluminescent HRP Substrate (Applygen Technologies, Beijing, China), and scans were obtained using the Bio-Rad gel image analysis system (BioRad, Hercules, CA, USA) and processed using Image-Pro Plus (Media Cybernetics, Inc.). The housekeeping protein glyceraldehyde 3-phosphate dehydrogenase (GAPDH; Bioworld Technology, Nanjing, China, 1:5000) was used as the loading control. 

#### 3.3.9. Statistical Analysis

All results are expressed as mean ± standard error (SEM). All statistical analyses were performed with SPSS software (version 16.0; SPSS Inc., Chicago, IL, USA) by Student’s t test, and one-way ANOVA with the next modified t-test. *p* values < 0.05 were considered statistically significant. The low number of animals and the many different statistical models may increase the risk of statistical errors.

## 4. Conclusions

Our results suggest that SCU prevents isoprenaline-induced myocardial fibrosis via inhibition of cardiac endothelial-mesenchymal transition potentially, which may be associated with the Notch pathway.

## Supplementary Materials

Supplementary materials can be accessed at: http://www.mdpi.com/1420-3049/19/10/15611/s1.

## Supplementary Files

Supplementary File 1
