# Supplementary material for: Anti-Fibrosis Effect of Scutellarin via Inhibition of Endothelial–Mesenchymal Transition on Isoprenaline-Induced Myocardial Fibrosis in Rats
Source: Molecules. 2014 Sep 29;19(10):15611–23. doi: 10.3390/molecules191015611 (PMC6271942; doi:10.3390/molecules191015611)
Supplement: Supplementary File 1 [file molecules-19-15611-s001.pdf]

Supplementary Materials

Table S1. Effects of scutellarin (SCU) on microvascular density (MVD) in an isoprenaline (Iso)-induced myocardial fibrosis rat model ($\bar{x} \pm s$). * $p < 0.05$, ** $p < 0.01$ *vs*. control group; ## $p < 0.01$ *vs*. Iso group.

Group	n	MVD
NS control	12	4.15 ± 0.77
Iso	14	1.80 ± 0.55 **
Iso + low dose SCU	12	3.95 ± 1.06 ##
Iso + high dose SCU	11	4.84 ± 0.82 ##*

Table S2. Fluorescence signals of α-sma and CD31 in the four groups ($\bar{x} \pm s$). Iso, isoprenaline; SCU, scutellarin; sma, smooth muscle actin; CD, cluster of differentiation. ** $p < 0.01$ *vs*. control group; # $p < 0.05$, ## $p < 0.01$ *vs*. Iso group.

Group	n	α-sma	CD31
NS control	14	0.11 ± 0.04	0.22 ± 0.10
Iso	16	0.23 ± 0.08 **	0.03 ± 0.03 **
Iso + low dose SCU	14	0.16 ± 0.08 #	0.11 ± 0.07 #
Iso + high dose SCU	15	0.12 ± 0.06 ##	0.23 ± 0.10 ##
